# Inactivation of dried cells and biofilms of *Listeria monocytogenes* by exposure to blue light at different wavelengths and the influence of surface materials

**DOI:** 10.1128/aem.01147-23

**Published:** 2023-10-17

**Authors:** Magdalena A. Olszewska, Govindaraj Dev Kumar, Minji Hur, Francisco Diez-Gonzalez

**Affiliations:** 1 Center for Food Safety, University of Georgia, Griffin, Georgia, USA; 2 Department of Industrial and Food Microbiology, The Faculty of Food Science, University of Warmia and Mazury in Olsztyn, Olsztyn, Poland; The Pennsylvania State University, State College, Pennsylvania, USA

**Keywords:** blue light, *Listeria*, disinfection, antimicrobial, surface treatment, biofilms

## Abstract

Antimicrobial blue light (aBL) in the 400–470 nm wavelength range has been reported to kill multiple bacteria. This study assessed its potential for mitigating an important foodborne pathogen, *Listeria monocytogenes* (Lm), focusing on surface decontamination. Three wavelengths were tested, with gallic acid as a photosensitizing agent (Ps), against dried cells obtained from bacterial suspensions, and biofilms on stainless-steel (SS) coupons. Following aBL exposure, standard microbiological analysis of inoculated coupons was conducted to measure viability. Statistical analysis of variance was performed. Confocal laser scanning microscopy was used to observe the biofilm structures. Within 16 h of exposure at 405 nm, viable Lm dried cells and biofilms were reduced by approx. 3 log CFU/cm^2^ with doses of 2,672 J/cm^2^. Application of Ps resulted in an additional 1 log CFU/cm^2^ at 668 J/cm^2^, but its effect was not consistent. The highest dose (960 J/cm^2^) at 420 nm reduced viable counts on the biofilms by 1.9 log CFU/cm^2^. At 460 nm, after 800 J/cm^2^, biofilm counts were reduced by 1.6 log CFU/cm^2^. The effect of material composition on Lm viability was also investigated. Irradiation at 405 nm (668 J/cm^2^) of cells dried on polystyrene resulted in one of the largest viability reductions (4.0 log CFU/cm^2^), followed by high-density polyethylene (3.5 log CFU/cm^2^). Increasing the dose to 4,008 J/cm^2^ from 405 nm (24 h), improved its efficacy only on SS and polyvinyl chloride. Biofilm micrographs displayed a decrease in biofilm biomass due to the removal of biofilm portions from the surface and a shift from live to dead cells suggesting damage to biofilm cell membranes. These results suggest that aBL is a potential intervention to treat Lm contamination on typical material surfaces used in food production.

**IMPORTANCE:**

Current cleaning and sanitation programs are often not capable of controlling pathogen biofilms on equipment surfaces, which transmit the bacteria to ready-to-eat foods. The presence of native plant microbiota and organic matter can protect pathogenic bacteria by reducing the efficacy of sanitizers as well as promoting biofilm formation. Post-operation washing and sanitizing of produce contact surfaces might not be adequate in eliminating the presence of pathogens and commensal bacteria. The use of a dynamic and harmless light technology during downtime and close of operation could serve as a useful tool in preventing biofilm formation and persistence. Antimicrobial blue light (aBL) technology has been explored for hospital disinfection with very promising results, but its application to control foodborne pathogens remains relatively limited. The use of aBL could be a complementary strategy to inactivate surfaces in restaurant or supermarket deli settings.

## INTRODUCTION

*Listeria monocytogenes* (Lm) is one of the major concerns for environmental contamination of ready-to-eat foods due to its widespread distribution and ability to survive well in the environment of packing- and processing-related surfaces and equipment (1). The caramel apple outbreak in 2014–2015 stressed the need for additional interventions because the grower’s packing process was the likely source of contamination as Lm was detected on the packing line and exposed porous surfaces were present (2). Similarly, investigating another outbreak linked to stone fruits identified the fruit packing facility as the contamination point (3). Listeriosis outbreaks in dairy foods continue to occur even in pasteurized Hispanic cheeses, which suggests environmental contamination. The most recent case linked the consumption of fresh Hispanic cheeses to 15 infections (4). These events justify the idea of developing novel alternatives for processing and packing environment treatments.

In recent years, the application of blue light has been explored and adapted for multiple purposes. In material sciences, ultraviolet (UV) and blue light lamps have been widely used for 3D printing for controlled photopolymerization of different synthetic materials (5). The use of blue light has also been adopted to enhance plant health and the nutritional quality of crops and medicinal plants (6). A recent study evaluated the effect of blue light on blueberries during cold storage to enhance food quality and shelf life (7). These multiple applications have rapidly developed flexible, low-cost, light-emitting diode (LED) technologies.

Ultraviolet light, particularly UV-C, which has a wavelength shorter than 280 nm, has been used as a disinfectant for many years in clinical, biomedical, laboratory, and food production (8). Its widespread utilization, however, is limited by its detrimental effects on the eyes and skin. Because UV light damages DNA, it can have mutagenic effects on human cells. A novel method capable of complementing other available interventions is the application of antimicrobial blue light (aBL). The aBL consists of safe, visible blue light in the spectrum of 400–470 nm (9). Within the range, the visible color gradually changes from purplish blue to blue from lower to higher frequencies. Thus, various terms such as violet-blue, violet light, or high-intensity narrow-spectrum light have also been used to refer to aBL.

Multiple studies have reported the antimicrobial effectiveness of aBL. Hope et al. reported aBL to be active against *Provotellaceae* (10). In another report, the growth of *Salmonella* and methicillin-resistant *Staphylococcus aureus* was suppressed by aBL at a 470-nm wavelength (11). Similarly, Abana and colleagues observed that aBL at 455 nm inhibited *Escherichia coli* growth (12). Another promising use for aBL was reported in aquaculture after the successful inactivation of fish pathogens, including *Vibrio anguillarum*, *Edwardsiella tarda,* and *Streptococcus* (13). In 2016, Halstead et al. reported the successful application of aBL against a range of nosocomial wound pathogens such as *Acinetobacter baumanii, Enterobacter cloacae, Pseudomonas aeruginosa, Enterococcus faecium,* and *Klebsiella pneumoniae* in liquid cultures and biofilms (14).

Due to its safety and applicability, aBL has been tested for environmental decontamination of hospital rooms (15, 16). It is an emerging technology for clinical and hospital decontamination (17). The potential effect of aBL against Lm has been reported by a few studies. The inactivation of Lm by aBL in liquid and agar media was first reported in 2012 in two separate studies achieving a 5 log CFU/mL reduction with 185 J/cm^2^ (18, 19). In another publication, Kim and colleagues observed that Lm viability was reduced by 2.1 log CFU/mL after receiving 486 J/cm^2^. It was also more susceptible to 405 nm aBL than *Staphylococcus* and *Bacillus* strains under refrigeration conditions (20). In the first study of aBL applied to control Lm in fresh produce, the count of inoculated cantaloupes treated with 1,210 J/cm^2^ at 405 nm was reduced by 3 log CFU/cm^2^ (21). These reports indicate that Lm is susceptible to aBL, and that this technology could be applied to its environmental control. However, most previous studies show this pathogen reduction in aqueous systems. To optimize the aBL as an intervention, we must improve our understanding of the effects of blue light and Lm cells, e.g., while drying on inert surfaces.

Because stainless steel (SS) is widely used in the food industry due to its inherent corrosion resistance, we aimed to determine optimal aBL conditions for decontaminating Lm from SS. For this purpose, we tested aBL against dried cells after spot inoculation on the SS and surface colonization, referred to as biofilms. Several parameters were tested, including wavelengths, emission dose/exposure time, and exogenous photosensitization effect. We have also compared the aBL efficacy against Lm on different packaging/processing-related surfaces. The biofilm disruption characteristics due to exposure to blue light were also evaluated.

## MATERIALS AND METHODS

### Bacterial strains and culture conditions

A five-strain mixture of *L. monocytogenes,* which included strains ATCC 19115 (human isolate), ATCC 19117 (sheep isolate), “Coleslaw” (from coleslaw), G1091 (from coleslaw), and 2011L-2624 (from cantaloupe outbreak), were used in this study and were obtained from our culture collection at the Center for Food Safety. The first three strains were selected because of their strong biofilm-forming ability (22). Each strain was activated from stock cultures by three successive 24-h transfers in 25 mL tryptic soy broth (TSB; Difco Laboratories, Sparks, MD, USA) supplemented with 0.6% yeast extract and incubated at 35°C for 24 h. After the final incubation, each of the five strains was spread plated on tryptic soy agar (TSA; Difco) with 0.6% tryptic soy agar with yeast extract (TSAYE) and placed in an incubator at 35°C for 24 h. The plate cultures were scraped off the agar using a sterile cell lifter and re-suspended into sterile phosphate-buffered saline (PBS; Sigma-Aldrich, St. Louis, MO, USA). The suspensions were diluted to attain a final concentration of 8 log CFU/mL and pooled to form a cocktail and used to inoculate different inert surface materials.

### Target material surfaces

SS, polyvinyl chloride (PVC), silicone rubber (SR), high-density polyethylene (HDPE), and polystyrene (PS) 25 mm × 75 mm coupons (Biosurface Technologies, Bozeman, MT, USA) were used to represent the constitutive materials of food contact surfaces, totes/bins, tarps, containers, pipes, and flooring.

### Dried cells

For dry cells, 100 µL of a mixed strain cocktail was inoculated onto the stainless-steel coupons in 20 spots and dried in a biosafety cabinet for up to 2 h. Coupons were prepared in duplicate, where one series included a photosensitizer (Ps) application and the other did not.

### Biofilms

SS coupons were used to obtain biofilms. Lying flat coupons were submerged into 50 mL TSB dispensed into sterile Petri dishes (Φ 150 mm) containing the cocktail (6 log CFU/mL) and incubated at 37°C for 48 h in an orbital shaker (100 rpm) to allow biofilms to be formed on the surface. Coupons were then washed with PBS to remove planktonic cells and dried as described above. They underwent photosensitization tests as dried cells did.

### Photosensitization

The efficacy of the evaluated light treatments included sublethal gallic acid concentration (10 mM; Thermo Fisher, Waltham, MA, USA). Gallic acid solutions were sprayed over the surface of the coupons as a mist (100 µL total volume) and immediately placed under the appropriate light source. 

### Blue light treatments

Commercially available LED arrays were tested at 405, 420, and 460 nm. The lamp descriptions are 405 nm LED array lamps (200 W, Fasttobuy), 420 nm LED array lamps (3 W, Xinhongye), and 460 nm LED array lamps (25 W, Byingo). Lights were placed 20 cm away from the coupons except for 420 nm, which needed a shorter distance due to its small size and power. Blue light exposures were conducted at selected time intervals (4, 8, and 16 h). A FieldMaxII-TO power meter with a PS19 power sensor (Coherent, Santa Clara, CA, USA) and a Mavospec Base spectrometer (Gossen, Nürnberg, Germany) were used to measure light intensity and peak wavelength, respectively. Emission doses as J/cm^2^ were calculated by measuring the light intensity with the radiometer and multiplying the W/cm^2^ values times the exposure time in seconds.

### Microbiological analysis

Treated coupons were placed in Fisherbrand sterile sampling bags containing PBS and transferred to an ultrasonic bath (60 Hz, VWR International, Radnor, PA, USA) for 2-min sonication to recover the cells. The contents were transferred into tubes and vortexed, and the viable cells in the PBS suspension released from coupons were serially diluted and spread in duplicate onto TSA plates. Petri plates were incubated at 37°C for 24 h, colonies were counted, and log CFU/cm^2^ was calculated.

### Confocal laser scanning microscopy

To study the biofilm disruption, a mixed strain culture was added to 8-well chamber slides (Nunc II; Lab-Tek; Fisher Scientific) to allow biofilms to be formed in the wells. After the development of biofilms, the wells were rinsed with NaCl (8.5 g/L) and refilled with NaCl, of which one set of wells was used as control, and another was exposed to blue light. Fluorescent labeling of the biofilms was carried out in the dark at room temperature for 20 min with a Syto 9 (5 μM), a green cell-permeant nucleic acid marker, and propidium iodide (20 µM, PI), a red impermeant nucleic acid marker (LIVE/DEAD BacLight Bacterial Viability Kit, Molecular Probes, LifeTechnologies, Eugene, OR, USA). After staining, the chambers were separated from the slides, and the biofilms submerged in the NaCl solution were thoroughly sealed underneath coverslips. Image acquisition was performed with a Zeiss LSM 700 Confocal Laser Scanning Microscope (Carl Zeiss Microscopy, Thornwood, NY, USA). All biofilms were scanned using a 40×/1.30 oil immersion objective lens with a 488-nm argon laser and a 555-nm diode-pumped solid-state laser. The fluorescence was recorded within the range from 500 to 600 nm to collect green fluorescence and from 610 to 710 nm to collect red fluorescence. Serial images were captured with a z-step of 1 µm, and three-dimensional projections of the biofilms were constructed using the Zeiss Zen 2.3 software (Carl Zeiss). Quantitative structural parameters were then extracted from confocal image series with COMSTAT 2, an image analysis software (www.comstat.dk) developed and described in references (23) and (24).

### Statistical analysis

Mean bacterial counts after each treatment were recorded as log CFU/cm^2^. All experiments were performed at least in two independent trials in triplicate. Significant differences among bacterial populations and biofilm density were determined using one-way analysis of variance. Principal component analysis (PCA) was performed to discriminate the influence of different factors on Lm cells. Before, cells were subjected to hierarchical cluster analysis to assess their similarity. Pearson’s correlation coefficients (*r*) were calculated to see if the substratum affected the Lm behavior. All statistical analyses were performed using Statistica software ver. 13.1 (StatSoft Inc., Tulsa, OK, USA). Differences were considered significant at a *P* < 0.05 level of probability.

## RESULTS

### Lm response to blue light on SS

The specific aims were to assess the aBL antimicrobial effect at three wavelengths at several doses, determine the influence of the application of a photosensitizer (gallic acid) and compare the effect on dried cells and biofilms.

**Fig 1:**
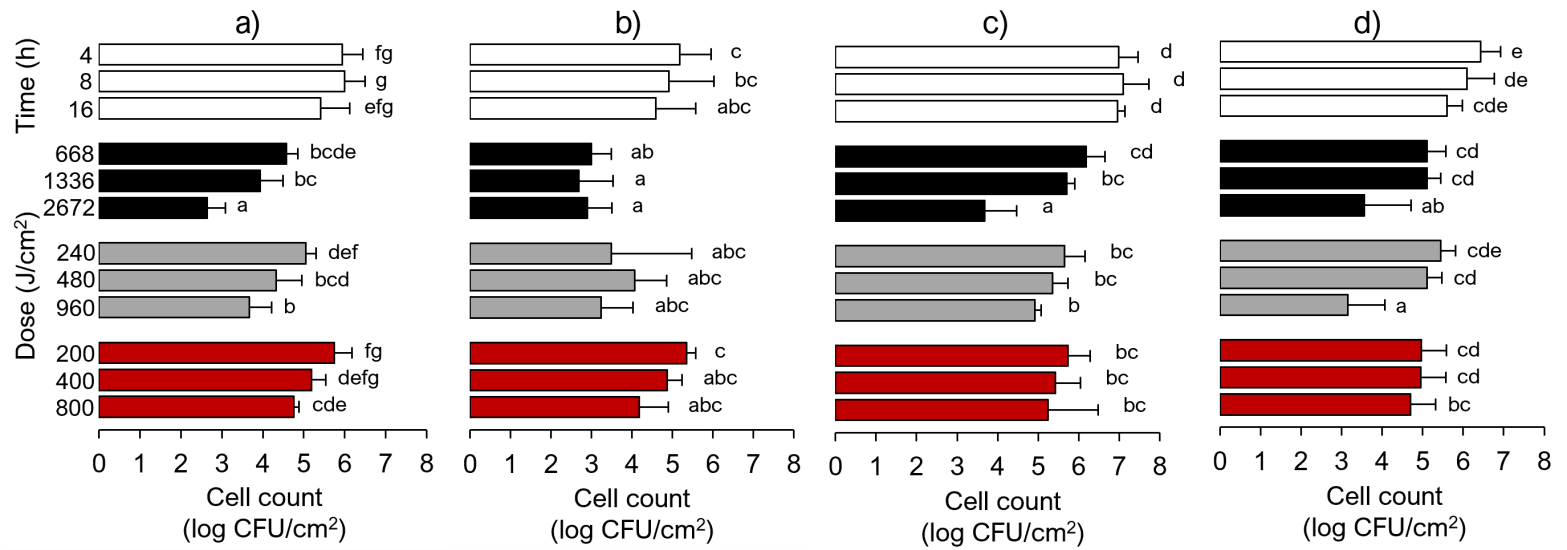
Effect of blue light on *Listeria monocytogenes* viability on stainless-steel coupons. (**a**) Dried cells, (**b**) dried cells with gallic acid as photosensitizer, (**c**) biofilms, and (d) biofilms with gallic acid. Graphs show controls (white bars), 405 nm (black bars), 420 nm (gray bars), and 460 nm (red bars). Control coupons were kept in the dark for the same period of exposure. Different letters indicate a significant difference at *P* < 0.05 within each cell type.

Treatments at 405 nm reduced the counts of dried cells inoculated onto SS coupons by 1.4, 2.1, and 2.8 log CFU/cm^2^ at 668 (4 h), 1,336 (8 h), and 2,672 J/cm^2^ (16 h), respectively (*P* < 0.05) (Fig. 1a). A gradual antimicrobial effect was also observed after exposing Lm to 420 and 460 nm. However, the final cell reductions did not exceed 2 and 1 log CFU/cm^2^ at 960 and 800 J/cm^2^, corresponding to 16-h exposures from 420 and 460 nm, respectively. The application of Ps resulted in an additional 1 log CFU/cm^2^ at 668 J/cm^2^, 4 h, from 405 nm (*P* < 0.05) (Fig. 1b). Ps enhanced the inactivation by 420 nm after the cells received the first dose of 240 J/cm^2^ within 4 h by an additional 0.8 log CFU/cm^2^; however, we did not find it statistically significant (*P* > 0.05). No such effect was found from 460-nm exposures.

We also tested the Lm wavelength sensitivity and exact emission doses/exposure times against biofilms (Fig. 1c). We confirmed the effectiveness of aBL at 405 nm against biofilms, where the 2,672 J/cm^2^ dose (16 h) caused 3 log CFU/cm^2^ reduction (*P* < 0.05) (Fig. 1c). However, biofilm cells survived better after receiving a lower dose than dried cells, including after Ps application. The cell reductions of only 0.8 and 1.3 log CFU/cm^2^ were observed at 668 J/cm^2^ (4 h). Sixteen-hour exposures from 420 and 460 nm significantly reduced Lm’s viability but did not exceed 2 log CFU/cm^2^ (*P* < 0.05). Also, Ps improved aBL efficacy by 420 nm but needed 16 h to enhance it by 0.5 log CFU/cm^2^ (*P* < 0.05) (Fig. 1d). Again, we did not observe any enhancement from Ps after 460-nm exposures.

**Fig 2:**
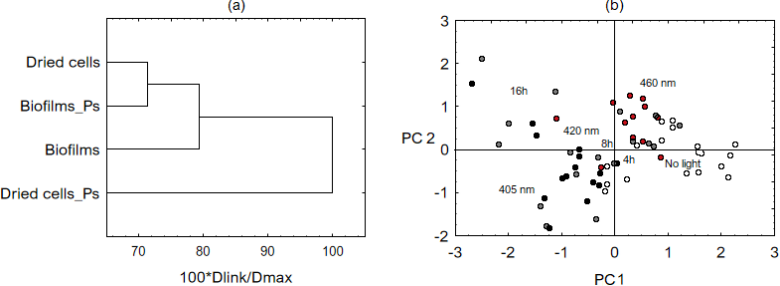
The dendrogram of a dissimilarity measure between the Lm cells (**a**). The principal component analysis biplot of light variables related to components 1 (PC1) and 2 (PC2) (**b**). Different wavelengths delivered different doses that cells received throughout 4-, 8-, and 16-h exposure time. Estimated doses were as follows: 668, 1,336, and 2,672 J/cm^2^ from 405 nm lamps (black dots); 240, 480, and 960 J/cm^2^ from 420 nm lamps (gray dots); 200, 400, and 800 J/cm^2^ from 460 nm lamps (red dots). Control coupons were kept in the dark for the same period of exposure (white dots).

PCA was performed to determine if the wavelengths or treatment conditions influence Lm viability on SS. Figure 2a demonstrates a dissimilarity between cells, where dried cells after the application of Ps are fused at a much higher distance than the remaining cell variants. As shown in Fig. 2b, the PCA biplot revealed two clusters, a collection of untreated/460 and 405 nm treated cells, with the 420 nm scattered between the two. It also separated exposure times corresponding to doses, with 16-h exposures at 405 and 420 nm plotting further away. It suggests longer irradiation decreases the Lm viability, especially following 405 and 420-nm exposures and applying Ps.

### Decontamination of inert surfaces

**Fig 3:**
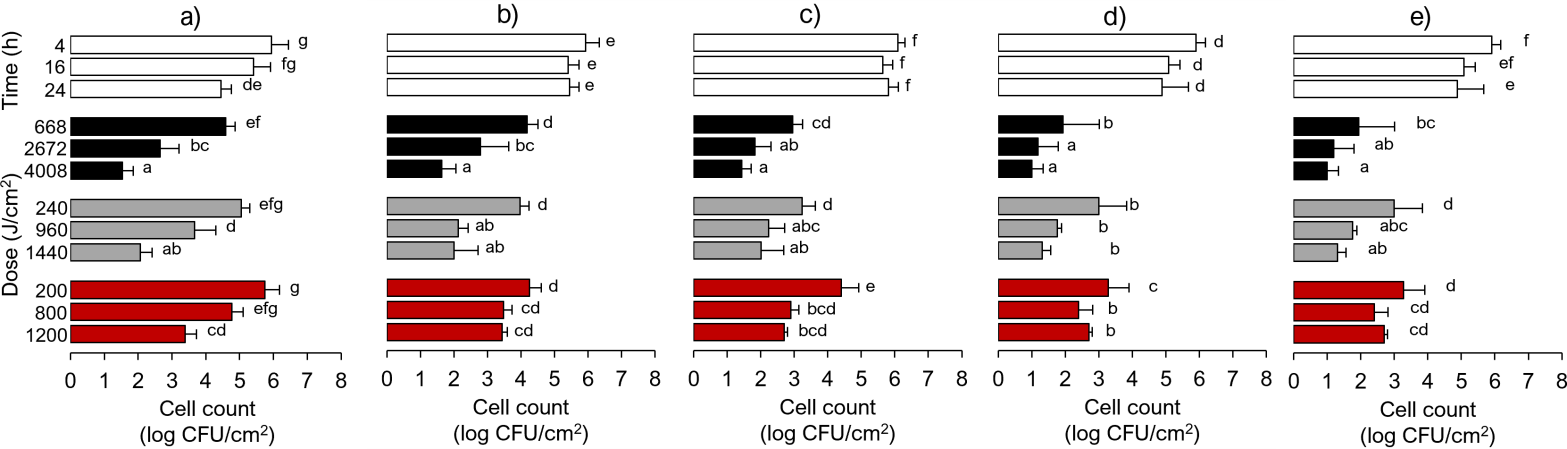
Effect of blue light on *Listeria monocytogenes* viability of cells dried on coupons made of different materials: (a) stainless steel, (**b**) polyvinyl chloride, (**c**) silicone rubber, (**d**) high-density polyethylene, and (e) polystyrene. Graphs show controls (white bars), 405 nm (black bars), 420 nm (gray bars), and 460 nm (red bars). Control coupons were kept in the dark for the same period of exposure. Different letters indicate a significant difference at *P* < 0.05 within each material.

The specific aim was to determine the influence of material composition on the reduction of Lm dried on the surfaces (Fig. 3). At 405 nm, the effectiveness of aBL varied depending on the surface material. At the lowest 405 nm dose (668 J/cm^2^, 4 h) reductions of 1.3, 1.8, 3.1, 3.6, and 4.0 log CFU/cm^2^ were observed on SS, PVC, SR, HDPE, and PS, respectively. However, those large differences among materials declined when final counts ranged between 0.8 and 1.6 CFU/cm^2^ after 4,008 J/cm^2^, 24 h. In control samples, interestingly, the viability of cells on PS and SS was reduced to 1.0 and 1.5 CFU/cm^2^, which was significantly greater than the viability on PVC, SR, and HDPE (*P* < 0.05).

Although smaller, significant cell reductions were observed after exposing Lm to 420 and 460 nm. Treatments at 420 nm resulted in 1.9 (240 J/cm^2^, 4 h) and 3.3 log CFU/cm^2^ (960 J/cm^2^, 16 h) from PVC and 2.9 and 3.3 CFU/cm^2^ from PS. Exposure to 460 nm caused the following reductions for the same two target materials: 1.7–1.9 log CFU/cm^2^ (PVC) and 2.6–2.7 CFU/cm^2^ (PS). Increasing exposure to 24-h exposures on any of the plastic/rubber materials did not result in significant reductions as compared to 16-h exposures. For surface comparison, Table 1 reveals their relationship strengths. For untreated coupons, the strongest linear relationship has been shown between SR and HDPE (*r* = 0.70). Other target materials scored either moderate or weak correlation. Following 4-h exposures, the *r* values increased between SR and HDPE (r = 0.79) and between them and SS, which was described as moderate. After 16 h, the correlation grew between SS and PS (*r* = 0.80). The weakest associations were noted for PVC, which paired with other target surfaces only after 24 h of exposure. The relationship between SR and HDPE remained strong and linear.

**TABLE 1:** Correlation coefficients (*r*) between target materials measure whether Lm survival to aBL was associated with the matrix type^*a*^

Treatment	Materials				
	SS	PVC	SR	HDPE	PS
No light					
SS					
PVC	**0.53**				
SR	0.14	**0.50**			
HDPE	0.33	**0.59**	**0.70**		
PS	**0.67**	**0.62**	0.37	0.45	
aBL 4 h					
SS					
PVC	0.05				
SR	**0.67**	0.33			
HDPE	**0.69**	0.21	**0.79**		
PS	**0.52**	0.16	0.44	0.43	
aBL 16 h					
SS					
PVC	0.34				
SR	**0.65**	0.24			
HDPE	**0.72**	0.01	**0.72**		
PS	**0.80**	0.46	0.50	**0.68**	
aBL 24 h					
SS					
PVC	**0.72**				
SR	**0.67**	**0.55**			
HDPE	**0.68**	**0.72**	**0.79**		
PS	**0.86**	**0.86**	**0.65**	**0.64**	

^*a*^ The correlation coefficients in bold are significant at *P* < 0.05. 0—no linear relationship. 0.3—a weak, positive relationship. 0.5—a moderate, positive relationship. 0.7—a strong linear relationship.

### Biofilm architecture and viability

**Fig 4:**
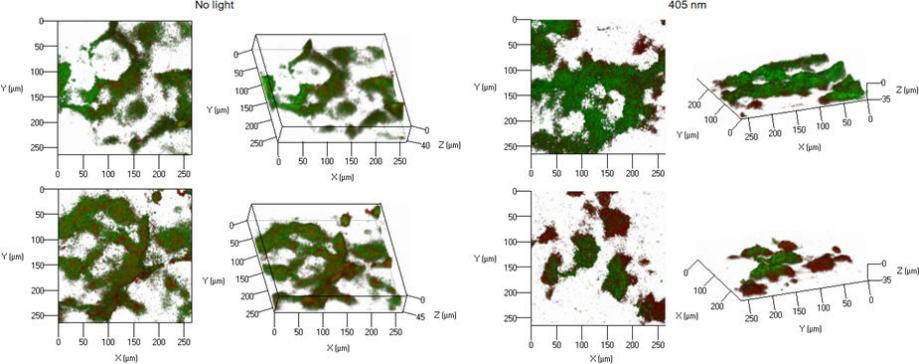
CLSM images of Lm biofilms treated with aBL at 405 nm (668 J/cm^2^) presenting transparent and rotated projections from random confocal z-stacks using ZEN 2.3 software. The biofilm was grown in an 8-well chamber slide system in tryptic soy broth for 48 h at 100 RPM and labeled with Syto 9 and propidium iodide, wherein cells were visualized based on cell membrane integrity.

The confocal laser scanning microscopy (CLSM) was conducted to determine shifts in the biofilm structure and cell membrane integrity within the first 4 h irradiation at all the wavelengths. LIVE/DEAD BacLight is an assay used to evaluate cellular viability based on the changes in membrane permeability. Green fluorochrome SYTO 9 penetrates intact and damaged cell membranes. In contrast, red fluorochrome PI can penetrate only damaged membranes, quenching SYTO 9 fluorescence. The images of stained biofilms are shown in Fig. 4. Nonirradiated biofilms demonstrated large green fluorescence, indicating that they were broadly viable. A few randomly distributed spots of cell death were noted, most likely due to regions of poor oxygen and nutrient content. In irradiated biofilms, several clusters revealed noticeable red fluorescence, indicating that these underwent a loss in the biological membrane integrity.

**Fig 5:**
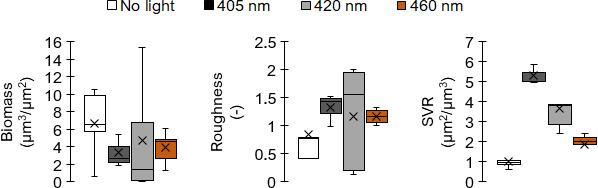
Box plots of the biofilm structural parameters: biomass, roughness, and SVR obtained for Lm after confocal data processing of Syto 9-labeled biofilms. The boxes range from the 25th to the 75th percentile and are intersected by the median line. Whiskers extend below and above the box range, from the lowest to the highest values, respectively. Averages are indicated by a cross symbol (×).

Next, biofilms’ biomass, roughness, and surface-to-volume values were extracted directly from confocal stack images since they were fairly easy to interpret in biological and physical terms (Fig. 5). Irradiated biofilms displayed a reduction in biofilm biomass due to the detachment of the biofilm portions and cells from the surface, which was confirmed by a higher roughness coefficient. After 405 nm, the biomass was relatively low, and the surface-to-volume (SVR) ratio was quite high. This corresponds to single cells and small cell clusters attached to the substratum. Single cells and small cell clusters naturally have a higher SVR than larger micro-colonies. Biofilms exposed to 420 nm scored second and to 460 nm scored third in terms of this parameter. It should be noted that a greater viability was observed when biofilms were treated at 420 nm as compared to 405 and 460 nm. We may speculate that this dispersion may be due to the relatively lower quality of 420 nm lamps.

## DISCUSSION

The possibility that the food packaging/processing-related surfaces may act as a reservoir of Lm is a serious concern and a significant source of food contamination. Therefore, effective mitigation of Lm from the food processing environment, including hard-to-clean sides, is essential. Compared to traditional chemical or UV disinfection, antimicrobial blue light has arisen as a novel approach to removing foodborne pathogens with non-toxicity and low microbial resistance. The response of Lm to aBL at different wavelengths, including after applying exogenous Ps, has become of interest to us. Although aBL may be an effective antimicrobial, much less is known about its effect on Lm cells deposited on inert surfaces, primarily stainless steel and on its biofilms.

The results indicated that the viability of Lm dried cells and biofilms was consistently reduced by exposure to all aBL wavelengths on SS. The Lm viability reduction depended on the dose, but it appeared to reach a maximum at a constant dose. Exposure to 405-nm blue light was more effective than treatment with 420 and 460-nm aBL lamps to inactivate cells seeded on the surface and biofilms. The viability of Lm, including in biofilms, was reduced by 3 log CFU/cm^2^ on SS after receiving 2,672 J/cm^2^, 16 h at 405 nm. Irradiation at 420/460 nm did not cause more than 2 log reductions, but cells received doses of no more than 1,000 J/cm^2^ within the same time. Biofilms were less susceptible to lower doses received within a shorter time, possibly due to hindered penetration across biofilm mass.

Most previous studies on irradiated SS demonstrated UV-C effectiveness in reducing bacterial biofilms, including Lm (25–28). Replacing it with blue light could be promising for surface decontamination. Lm is relatively frequent on surfaces of working spaces of the food industry, such as floors, drains, and specific equipment, even if these are often cleaned and disinfected (29). Also, Lm adheres to surfaces, forming biofilms resistant to common disinfectants and representing a significant concern for the food industry (30). For blue light to be effective against biofilms on a surface like SS, a 3-log reduction requires much longer exposure times. Using relatively low-intensity lamps, we could achieve it within 16 h of exposure at 405 nm.

This study also considered material composition on Lm viability to assess if aBL has the potential for different surface decontamination applications. Irradiation at 405 nm caused the most significant Lm reduction on polystyrene (4.0 log CFU/cm^2^) with the lowest dose of 668 J/cm^2^, followed by high-density polyethylene (3.5 log CFU/cm^2^), and silicone rubber (3.1 log CFU/cm^2^), suggesting a potential impact of type of materials on Lm inactivation from the surfaces of hydrophobic nature. Comparatively, this was not the case for PVC, which stood out as a material causing the least Lm reduction, thus questioning the modifications of PVC. This could be achieved by grafting polydimethylsiloxane and making it a superhydrophobic surface (31).

Also, some enhancement in Lm reduction was observed on SS or PVC after extending the 405-nm exposures to 24 h, which delivered 4,008 J/cm^2^. This was not the case for other wavelengths confirming that 16-h exposures attained consistent Lm inactivation. Still, 420 nm also appeared promising since it allowed us to reach larger Lm reductions than 460 nm from most target materials.

The use of photosensitizers can further improve the efficacy of aBL, especially on hard-to-reach surfaces. Although Lm has shown some sensitivity to Ps, gallic acid’s enhancing blue light inactivation was encouraging at 405 nm. Its application resulted in an additional 1 log reduction at 668 J/cm^2^ against dried cells. Previous studies showed that using gallic acid with light at 365 nm increased H_2_O_2_ production on tomato and lettuce surfaces, resulting in antimicrobial activity against *Salmonella* Newport (32). Gallic acid (4 mM) combined with blue light at 405 nm resulted in a 5 log CFU/cm^2^ reduction of *Staphylococcus aureus* (33). Similar antimicrobial activity was reported when aminolaevulinic acid was used with blue light against *Staphylococcus* (34). For the first time, we saw the potential of gallic acid as an exogenous photosensitizing agent at 420 nm, including for biofilms. Previous research supports the rationale of using exogenous photosensitizers such as curcumin, a powerful Ps, against biofilms. Bonifácio et al. (35) tested the combination of curcumin with blue light against *Listeria innocua* biofilms and achieved biofilm cell reductions of 5 logs. The inactivation efficiency attained with this natural compound was higher than with porphyrin.

The exact mechanism by which aBL causes bacterial cell death is unknown, but it has been hypothesized that it involves a photochemical effect. It has been suggested that the interaction of blue light with endogenous photosensitizing compounds, such as porphyrins and flavins, triggers the generation of reactive oxygen species. Blue light may induce photosensitizing molecules into an excited state, leading to an energy transfer to molecular oxygen. The excited oxygen may damage the cell integrity and thus induce cell death (36). The different bacterial sensitivity to the treatment could be in variations in the amounts and the types of these endogenous compounds present in bacterial cells.

O'Donoghue et al. (37) reported that Lm senses blue light via the flavin mononucleotide-containing sensory protein Lmo0799, which activates the general stress response sigma factor SigB (σ^B^). Kim et al. (20) suggested that the antibacterial mechanisms of blue light on Lm might be due to physical damage to bacterial membranes. To our knowledge, it has not been demonstrated on Lm biofilms. This study evaluated the wavelength sensitivity of Lm in biofilms after 4-h exposures. Irradiation of mature biofilm with blue light reduced biofilm biomass due to the removal of biofilm portions from the surface. The biomass detached from the surface and washed away was observed regardless of the wavelength applied. The ones that remained on the surface were stained with PI. Damage to cell membranes may result from losing their physiological functions, such as permeability barrier, membrane potential, or efflux pump activity. Our results imply that the treatments distorted the permeability of cell membranes since PI with high molecular weight can only go into the cells with the loss of membrane function as a permeability barrier (38). Similar cytoplasmic membrane damage was observed in *Pseudomonas aeruginosa* biofilms exposed to the oxidizing effects of chlorine (39) or blue laser (20). Thus, free radicals generated by blue light could physically or chemically damage the components of the bacterial membrane.

The scope of this work did not include an assessment of an actual application into a processing facility. This research used a set of commercially available lamps designed to deliver relatively low-intensity emissions of no more than 200 mW/cm^2^ for experimental purposes. The time of exposure was not a recommendation for the time that it would take if the technology were deployed. The exposure times were selected to be able to deliver larger emission doses. A potential use of aBL would likely employ industrial lamps capable of emitting more than 2,000 mW/ cm^2^, which will markedly shorten the exposure time.

### Conclusion

This study gives considerable evidence of the ability of the blue light to exert antimicrobial activity, with and without the addition of an exogenous photosensitizing agent, against the critical human pathogen Lm. While a few previous studies have addressed some aspects of this effect, mainly in aqueous systems, this work examined the wavelength sensitivity of Lm on surfaces. It appears particularly important for blue light’s rational and evidence-based applicability to control Lm in food-processing environments.
